# Diversification of cichlids in the genus *Danakilia* in the *D*anakil depression of Eritrea and Ethiopia

**DOI:** 10.1186/s12862-026-02509-9

**Published:** 2026-04-02

**Authors:** S. Elizabeth Alter, Giorgio Chiozzi, Giuseppe De Marchi, Mauro Fasola, Naoko P. Kurata, Melanie L. J. Stiassny

**Affiliations:** 1 Department of Biology, Agriculture and Chemistry, California State University Monterey Bay, Seaside, CA USA; 2 Department of Ichthyology, American Museum of Natural History, New York, New York USA; 3 Museo di Storia Naturale di Milano, Corso Venezia 55, 20121 Milan, Italy; 4 Società Italiana di Scienze Naturali, Corso Venezia 55, 20121 Milan, Italy; 5 Dipartimento di Scienze della Terra e dell’Ambiente, Università degli Studi di Pavia, Via Ferrata 1, 27100 Pavia, Italy; 6 Department of Natural Resources and the Environment, Cornell University, Ithaca, New York USA

**Keywords:** Population genomics, Phylogeography, RADseq, Extreme environments

## Abstract

**Background:**

Cichlids in the genus *Danakilia*, endemic to the hyper-arid Danakil Depression of Eritrea and Ethiopia, represent a rare example of diversification in one of Earth’s most extreme aquatic environments. While previous mitochondrial and morphological studies have identified two species (*D. franchettii* and *D. dinicolai*), the evolutionary history of this clade remains poorly resolved. Here, we use genome-wide SNP data from 85 individuals of *Danakilia*, sampled across six sites to infer phylogenetic relationships, assess population structure, and explore the role of historical hydrology in shaping diversification within the genus. We analyzed double-digest RAD-seq data to generate maximum likelihood phylogenies, PCA, and admixture profiles, and calculated pairwise FST values to assess genetic differentiation.

**Results:**

Our results support strong phylogenetic and population-level differentiation between the two described species and three additional riverine populations, with four to five discrete genetic clusters identified. Phylogenetic analyses confirm the monophyly of *D. dinicolai* and reveal that all riverine populations form a well-supported clade sister to *D. dinicolai*, suggesting a shared origin. In contrast, *D. franchettii* is not monophyletic, and populations from southern spring habitats near Lake Afrera show limited genetic structure. Patterns of genetic divergence are broadly consistent with a paleohydrological model in which a mid-Holocene lake system connected the northern and southern Danakil Depression, facilitating gene flow among now-isolated habitats. We also detect varying levels of admixture among the northern populations, with evidence of historical gene flow between the crater lake and riverine populations. Despite their close geographic proximity, the three riverine populations are genetically distinct and may represent independent evolutionary units.

**Conclusions:**

These findings suggest a complex interplay of historical connectivity, geographic isolation, and adaptation to extreme environmental gradients in driving divergence within *Danakilia*. Given emerging environmental threats in the region, our results underscore the urgent need for conservation attention and further exploration of unsampled populations to fully characterize the evolutionary and ecological diversity of this little-known lineage.

## Background

Cichlid fishes (Percomorpha, Cichlidae) are a model clade in evolutionary biology due to their history of rapid diversification across many freshwater environments, both in African lakes and extreme habitats including turbulent rapids [1, 2], deep lakes or rivers [3–7], and hot and/or alkaline spring systems [8, 9]. However, even among these extreme environments colonized by cichlids, perhaps the most challenging is the Danakil Depression of Ethiopia and Eritrea, a seismically active part of the Great Rift Valley [10]. Previously a gulf of the Red Sea during the last interglacial (128-117kya), the Danakil Depression has experienced dramatic changes in water level over its geological history that have occasionally connected the northern and southern end of the depression. Today, these two areas are separated by a sill that sits at about −80 m asl (unpublished results). Temperatures in the Danakil are among the highest on the planet, with recorded annual averages up to 34.7 °C [11]. Precipitation is negligible (<100 mm/year), and evaporation rates are high (up to 5 m/year [12, 13]), with most lake waters characterized by hypersaline conditions [14, 15]. Yet despite such harsh conditions, some spring-fed lakes and a few creeks in the Danakil Depression harbor two described species and several potentially undescribed lineages in the endemic genus *Danakilia* (Cichlidae, Oreochromini) [16, 17].

Little is known about the history of diversification of *Danakilia*, which has likely been driven by a combination of geohydrology, selective filters, rapid adaptation, and allopatric divergence across isolated water bodies. Addressing this knowledge gap will provide an improved understanding of cichlid evolution in extreme habitats. Additionally, resolution of the origins and taxonomic status of distinct lineages within *Danakilia* have taken on added urgency with the increasing extraction of salt from Lake Afrera and the impending development of extensive potash mining throughout the Danakil (and anticipated major impacts on fragile aquatic habitats).

**Fig. 1:**
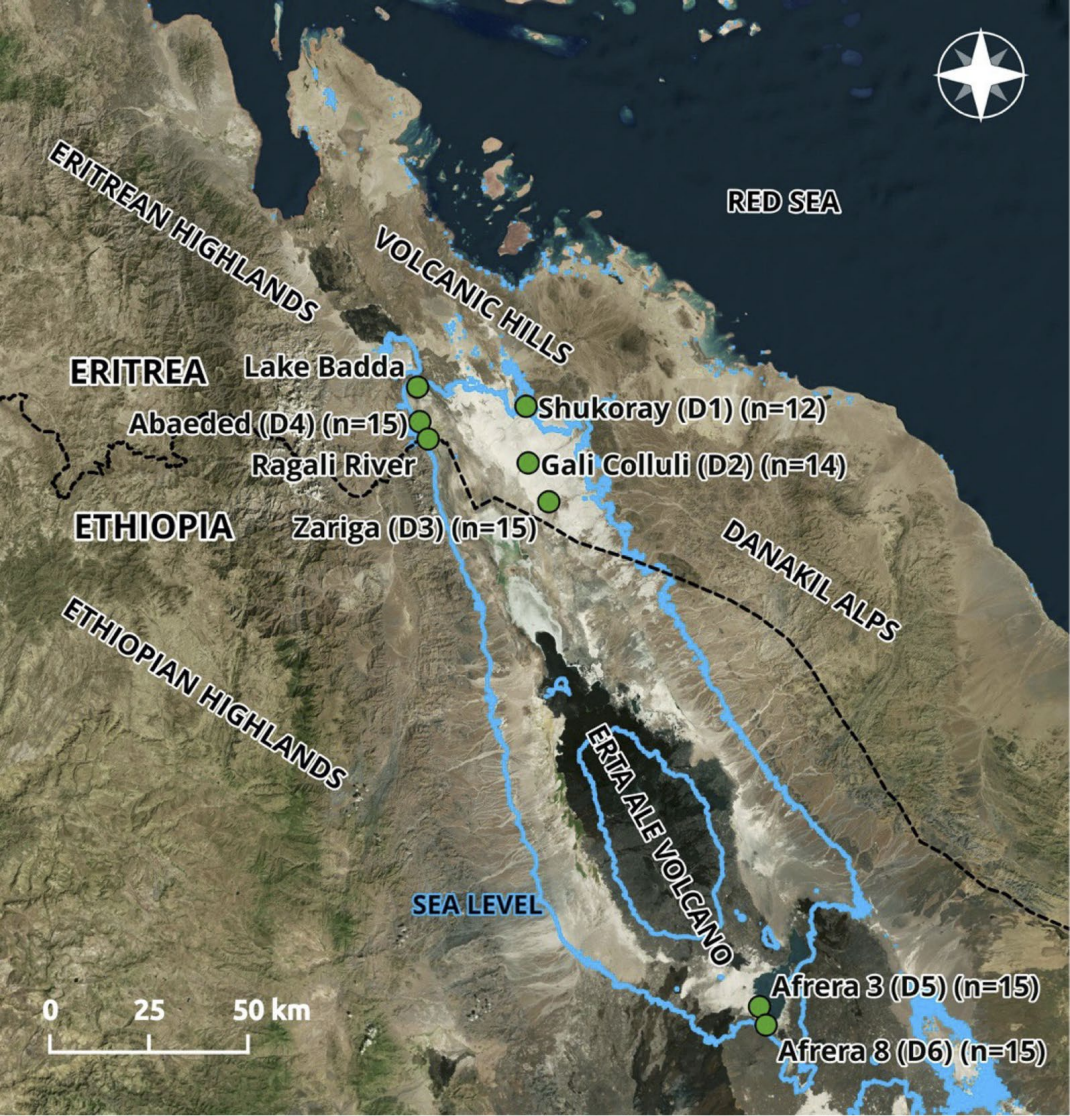
Map of the study area with the location (green dots) of the collection sites (*n* = sample size). The light blue lines delineate the areas below sea level, referred to as the Danakil depression. Samples collected in Lake Abaeded (sampling site D4) fall within the range of the previously described species *D. dinicolai* whereas samples collected in Afrera 3 and Afrera 8 (D5, D6) correspond to the range of *D. franchetti*

While the detailed evolutionary history of the genus *Danakilia* is unclear, morphological evidence support inclusion in the tribe Oreochromini [18] and a recent genetic analysis identified a close relationship with the oreochromin *Iranocichla* from southern Iran [19]. However, as no close relatives of *Danakilia* have been identified in the Awash or Nile Rivers (the closest systems to the Danakil Depression), the origin of this clade in the region remains uncertain. Currently two valid species are recognized: *Danakilia franchettii* [20] and *D. dinicolai* [21]. *Danakilia franchettii* is found exclusively in the southern end of the depression, where it inhabits streams and pools originating from hot springs feeding Lake Afrera (also called Afdera) but is absent from the lake itself [20, 22] (Fig. 1). The second species, *D. dinicolai*, was described from the small volcanic Lake Abaeded, roughly 170 km north of the range of *D. franchettii* [21]. Additional populations of *Danakilia*, the taxonomic status of which remains unclear, have recently been found inhabiting three ephemeral creeks (Shukoray, Gali Colluli, and Zariga) in the northern part of the Danakil Depression near Lake Abaeded. An additional population has been reported from the Ragali River, the course of which partially marks the Eritrean and Ethiopian border (Andrew Hickman and Andrew Cauldwell, pers. comm.) but military activities in that region precluded investigation of this lineage.

Two previous studies have examined relationships between lineages within *Danakilia*. In the first population-level genetic analysis of the genus, Chiozzi et al. [16] used phylogeographic patterns of two mitochondrial DNA markers (COI and cytochrome *b*) and geological reconstructions to investigate differences between putative lineages. That analysis recovered genetic separation between the two species but no differentiation between *D. dinicolai* populations in Lake Abaeded and the three riverine populations. The geological reconstructions of Chiozzi et al. [16] suggested that during the mid-Holocene (9800–7300 YBP), water levels reached up to 50 m above present levels [23], creating a massive paleo-lake across much of the depression, which likely facilitated ancestral population expansion and potential gene flow across the entire region. This framework hypothesizes that a dramatic reduction in water level beginning roughly 7300 YBP isolated populations in southern and northern regions of the depression.

A subsequent morphological study comparing body and lower pharyngeal jaw shape [17] found significant differences between the southern *D. franchettii* and northern *D. dinicolai* (plus riverine) populations, but only minor differences between the three riverine populations and *D. dinicolai* of Lake Abaeded. While subtle, the degree of morphological variation described was suggestive that these populations may be on independent evolutionary trajectories, and could constitute separate evolutionary units important for regional conservation planning.

While these two studies greatly improved understanding of this recently diverged clade, illumination of its phylogeographic history requires more extensive genome-wide data. Therefore, to better explore the diversification history of *Danakilia* we collected SNP data using ddRAD-seq [24]. We analyzed genomic diversity and demographic histories across populations of *Danakilia,* including the two described lake species, *D. dinicolai* and *D. franchettii*, and three riverine populations spanning the known range of the genus (exclusive of the reported Ragali River population). We used these data to 1) delimit and assess the number of discrete lineages within *Danakilia* and relate these to morphological differentiation and biogeographic patterns; 2) determine phylogenetic relationships between lineages; and 3) examine the history of hybridization/admixture. Specifically, we assessed whether the three riverine populations, the population of *D. dinicolai,* and two populations of *D. franchettii* represent independent evolutionary lineages and examined the evidence for past and current gene flow between these populations.

## Methods

### Sample collection and DNA extraction

We collected tissues from a total of 85 specimens of *Danakilia* from six sites in the Danakil depression during 2014–2016, across three joint expeditions organized within the framework of collaborations among the University of Pavia (Italy), the American Museum of Natural History (USA), the Ministry of Marine Resources of Eritrea and the Ethiopian Biodiversity Institute. These expeditions revealed three new populations of *Danakilia* (Table 1: Rivers Shukoray, Gali Colluli, Zariga). Chiozzi et al. [16, 17] provide details on differences in the male breeding coloration, shape variation, and mitochondrial diversity in these populations compared with *D. dinicolai* and *D. franchettii*. The six sampling sites included: the ephemeral rivers Shukoray (population code D1), Gali Colluli (D2), Zariga (D3) in the northeastern portion of the study area, in the crater lake Abaeded (D4) in the northwest, and two springs that feed Lake Afrera in the south: Afrera “spring 3” (D5) and Afrera “spring 8” (D6) (Fig. 1, Table 1). After collecting individuals using seine and hand nets, fin clips were taken using sterilized tools and preserved in 95% ethanol (fish were anaesthetized with eugenol before fin clips were obtained, kept under observation until they had fully recovered, then released back into their environment; details regarding handling of individuals are presented in [16]). In addition, we included fin clips from seven individuals from the related cichlid genera *Iranocichla* and *Sarotherodon*, obtained from the AMNH ichthyological collection, to represent outgroups. Genomic DNA was isolated from fin clips using DNeasy Blood and Tissue Kit (Qiagen) according to the manufacturer’s protocol.

**Table 1:** Number of individuals sampled per location and genetic diversity across variant and fixed sites. Site D4 corresponds to the range of the previously described species *D. dinicolai* and sites 5/6 correspond to *D. franchetti*. SG= *Sarotherodon boulengeri*, IH= *Iranocichla hormuzensis;* H = observed heterozygosity; π=average nucleotide diversity. No values for H and π are reported for SG and IH as these species are included as outgroups and the specimens included do not represent populations

Population code	Locality	#samples	H	π
D1	River Shukoray, Eritrea	12	0.02447	0.06895
D2	River Gali Colluli, Eritrea	14	0.02544	0.06595
D3	River Zariga, Eritrea	12	0.02898	0.07114
D4	Lake Abaeded, Eritrea	15	0.02662	0.07029
D5	Lake Afdera, Spring 3, Ethiopia	15	0.0306	0.0684
D6	Lake Afdera, Spring 8, Ethiopia	5	0.02868	0.07104
SG	Democratic Republic of the Congo	3	n/a	n/a
IH	Rudan River and Mehran River, Iran	4	n/a	n/a

### Library preparation and sequencing

We collected genome-wide SNP data using the double-digest restriction amplification protocol described by Peterson et al. [24]. Briefly, we first digested 500ng of genomic DNA with enzymes NlaIII and MluCI (New England Biolabs) at 37 °C for 5 hours. Following a magnetic bead purification (Axygen), DNA was ligated to barcodes and Illumina adapters, and samples were pooled in equimolar concentrations. We selected a size range of 300-450bp fragments (including adapters) for sequencing using a Blue Pippin Prep (Sage Sciences), and ligated Illumina indexing primers to these fragments. We assessed the size distribution and final DNA concentrations of these fragments using an Agilent 2200 and qPCR, and sequenced fragments on an Illumina HiSeq 4000 (PE, 2x150bp) across four lanes with 10% PhiX spike-in.

### Data analysis

We used FastQC v0.10.1. to assess the overall quality of the dataset, and employed the ipyrad pipeline [25] (https://github.com/dereneaton/ipyrad)) to trim read ends and adapters, perform reference-based assembly, and call SNPs. The closest and the most recent available genome of *Oreochromis niloticus* (O_niloticus_UMD_NMBU) was used as the reference. The ipyrad pipeline was performed with the following key parameters: phred_Qscore_offset was set to 43; adapter filtering was enabled (filter_adapters = 3), and reads were trimmed to a minimum length of 35 bp (filter_min_trim_len = 35). Loci were retained if they were present in at least four samples (min_samples_locus = 4), with a minimum depth of 6 for statistical base calling (mindepth_statistical = 6) and 1 for majority rule (mindepth_majrule = 1). Clustering was performed at a similarity threshold of 85% (clust_threshold = 0.85). Other filtering thresholds included a maximum of 5 low-quality bases per read, maximum read depth of 10,000 (maxdepth = 10000), and no barcode mismatches allowed (max_barcode_mismatch = 0). Consensus base calling parameters were set to allow up to 2 alleles (max_alleles_consens = 2), 5% ambiguous bases (max_Ns_consens = 0.05), and 5% heterozygous sites (max_Hs_consens = 0.05). Additionally, loci were excluded if they contained more than 20% SNPs (max_SNPs_locus = 0.2), more than 8 indels (max_Indels_locus = 8), or if more than 50% of individuals shared heterozygous sites at a locus (max_shared_Hs_locus = 0.5).

*Phylogenetic analysis.* We used maximum-likelihood methods in IQ-Tree [26] to infer the evolutionary relationships between lineages, using a final dataset comprised of all samples and loci that were retained after applying the criteria described above. Seven individuals from related cichlid genera were used as outgroups (four individuals of *Iranocichla hormuzensis*, and three individuals of *Sarotherodon boulengeri*). We implemented 1000 aLRT replicates (−alrt 1000) and 1000 ultrafast bootstrap replicates (−bb 1000) to obtain confidence values for each node, with auto-estimation of number of CPUs (−nt AUTO). ModelFinder Plus (implemented in IQ-Tree as -m MFP) was used to determine the best evolutionary model.

*Analysis of genetic diversity, population structure and admixture* (PCA, sNMF, Fst values). Observed heterozygosity and nucleotide diversity (π) were calculated using the ipyrad-analysis toolkit. Given the young age of the genus, we used the R package PCADAPT [27] to generate a Principal Components Analysis (PCA) to explore population-level diversity and structure within lineages. We also used the R package sNMF [28] to assess shared ancestry between populations. Using sNMF we tested K values from 1 to 6 with regularization parameters alpha of 1, 10, 100, and 1000. The data were diploid, and cross-entropy was calculated to evaluate model fit. Each K–alpha combination was replicated 10 times to account for random initialization, and computation was parallelized using 10 CPU threads.

## Results

### Sequencing and bioinformatics

Sequencing ddRAD fragments on an Illumina HiSeq platform recovered 150,744,934 raw reads. After quality filtering, 147,586,259 reads remained. One individual (D3_7) was removed due to having < 100,000 raw reads. SNPs were filtered using VCFtools [29] to retain only biallelic loci present in ≥70% of individuals (i.e., ≤30% missing data). The filtered dataset comprised 84 Danakilia individuals and 13,129 SNP loci, and was used for phylogenetic inference; all retained individuals had data for >56% of loci. For population structure and admixture analyses, we further reduced the dataset by retaining a single SNP per RAD locus to minimize linkage disequilibrium, resulting in 3520 putatively unlinked loci, with individuals retaining data for >51% of loci.

### Inference of phylogenomic history

**Fig. 2:**
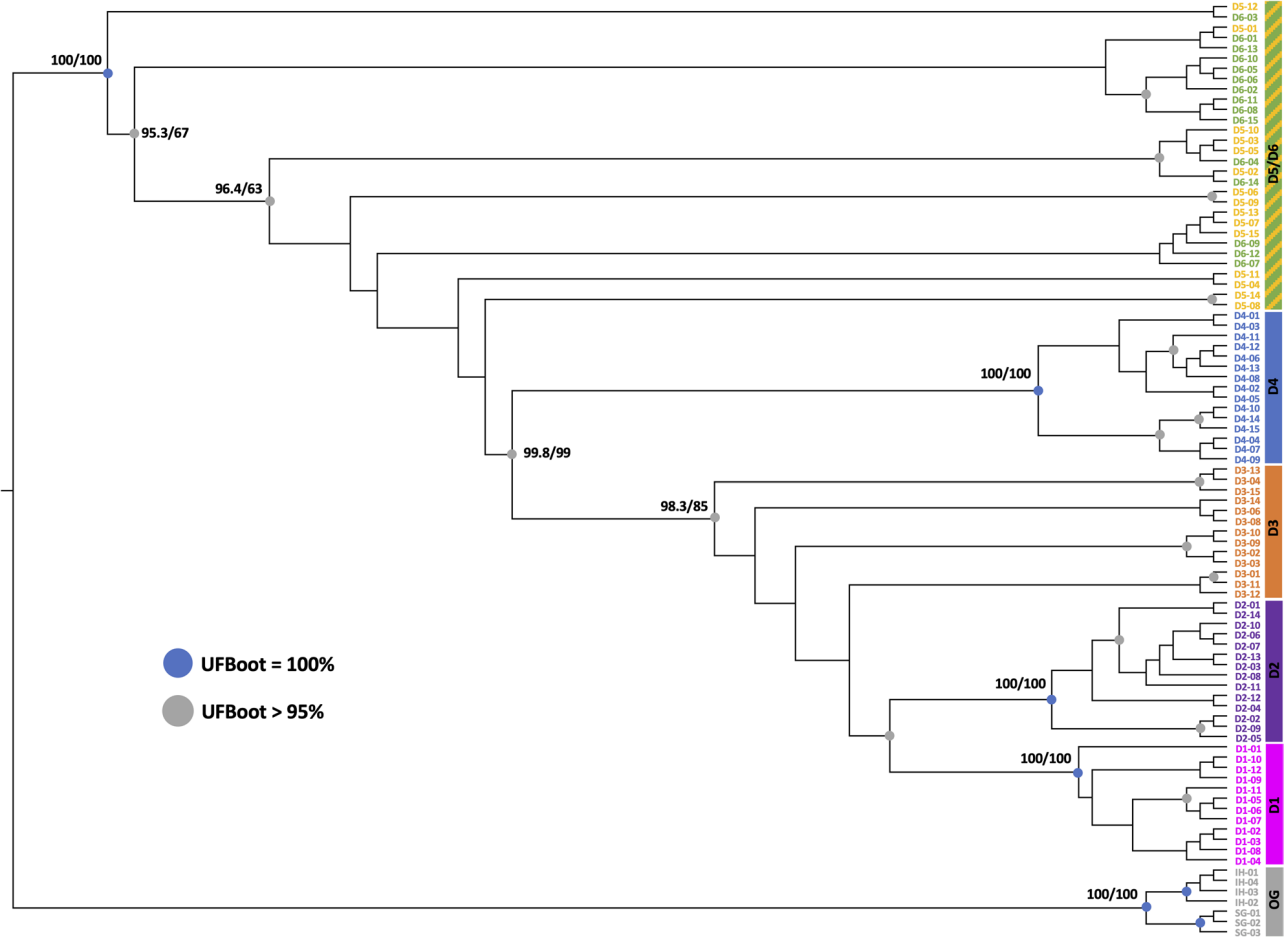
Phylogenetic relationships (maximum likelihood tree inferred in IQTree; branch support values estimated via 1000 bootstrap replicates of ultrafast bootstrap approximation (UFBoot) and SH-like approximate likelihood ratio test (SH-aLRT). Literature suggests generally robust values for these supports would be UFBoot > 95% and SH-aLrt > 80%. D1: shukoray, D2: Gali Colluli, D3: Zariga, D4: abaeded (*D. dinicolai*), D5: Afdera3 (*D. franchetti*), D6: Afdera8 (*D. franchetti*). Blue circles represent sampling locations/populations that are monophyletic and have 100% bootstrap support (D1, D2, D4); gray circles represent clades with >95% bootstrap support

IQ-Tree was used to determine a best-fit substitution model of TVM+F+R2 for the dataset and to infer evolutionary relationships, including all SNPs per RAD locus (i.e., not pruned for linkage) and <30% per-individual missing data (CPU time used for tree search: 57,965.302 sec). The inferred tree shows strong support for monophyly for the genus as a whole (Fig. 2). In addition, monophyly is strongly supported for *D. dinicolai* (D4) originally described from Lake Abaeded. All three of the riverine populations cluster in one well-supported (bootstrap values UFBoot/SH-aLRT = 98/99) clade that groups with *D. dinicolai* of Lake Abaeded (99.8/99). The Shukoray (D1) river population is also monophyletic with strong support (100/100), as is Gali Colluli (D2) (100/100). However, *D. franchettii* (D5, D6) is not recovered as monophyletic, nor are samples from the Zariga river (D3). No phylogenetic structure is apparent between the two sampling locations from springs adjacent to Lake Afrera (D5, D6). Tree topology indicates the multiple lineages found near Afrera appear to have diverged prior to divergence of clades in the northern depression (Abaeded and riverine clades). In addition, the tree topology suggests the Shukoray and Gali Colluli populations diverged after the population(s) in Zariga.

### Genetic diversity and gene flow

**Fig. 3:**
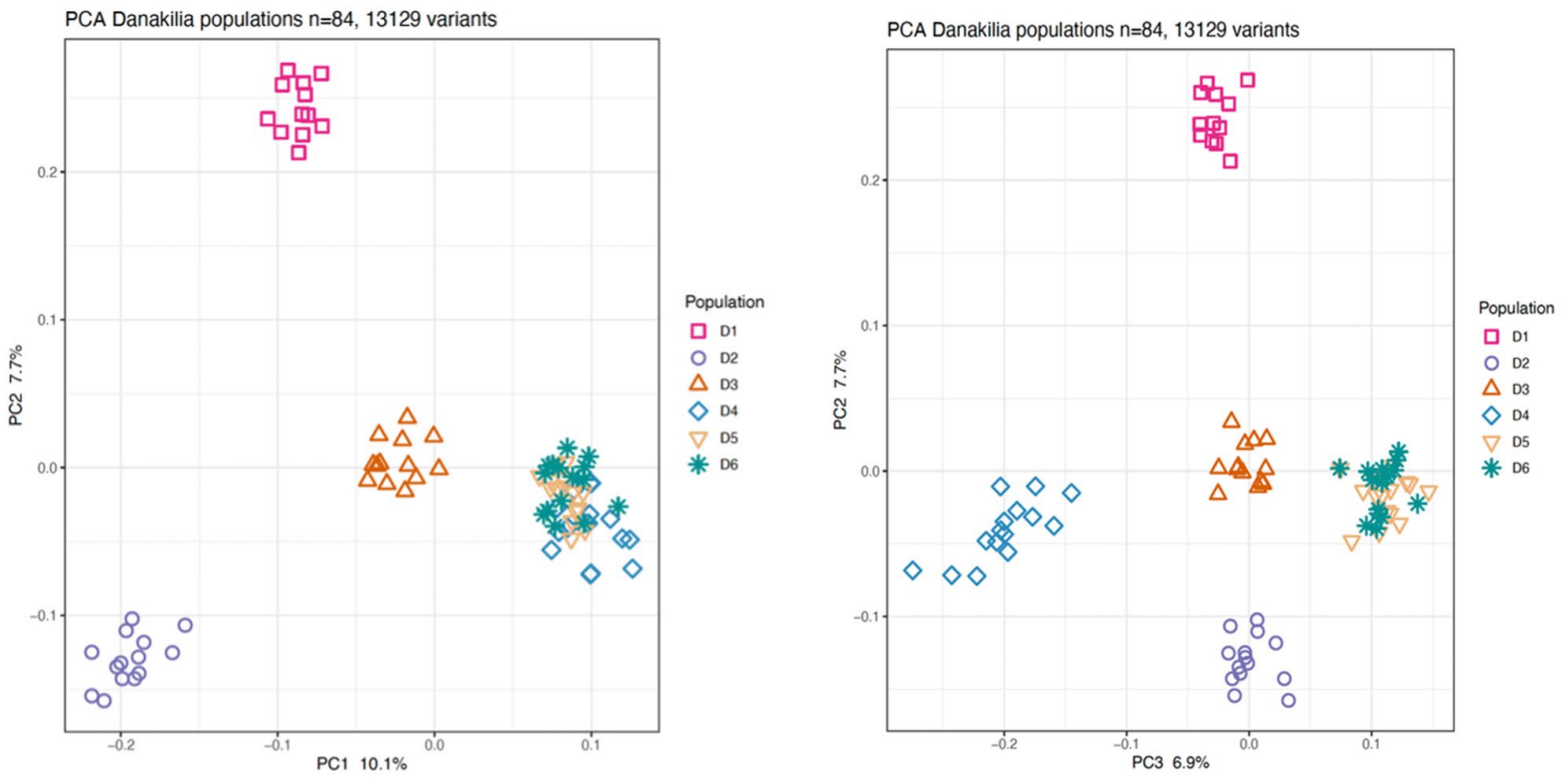
Population-level PCA (*Danakilia* only, without outgroup): D1: shukoray, D2: Gali Colluli, D3: Zariga, D4: abaeded (*D. dinicolai*), D5: Afdera3 (*D. franchetti*), D6: Afdera8 (*D. franchetti*). (**a**) PC1 v PC2; (**b**) PC2 v PC3

**Fig. 4:**
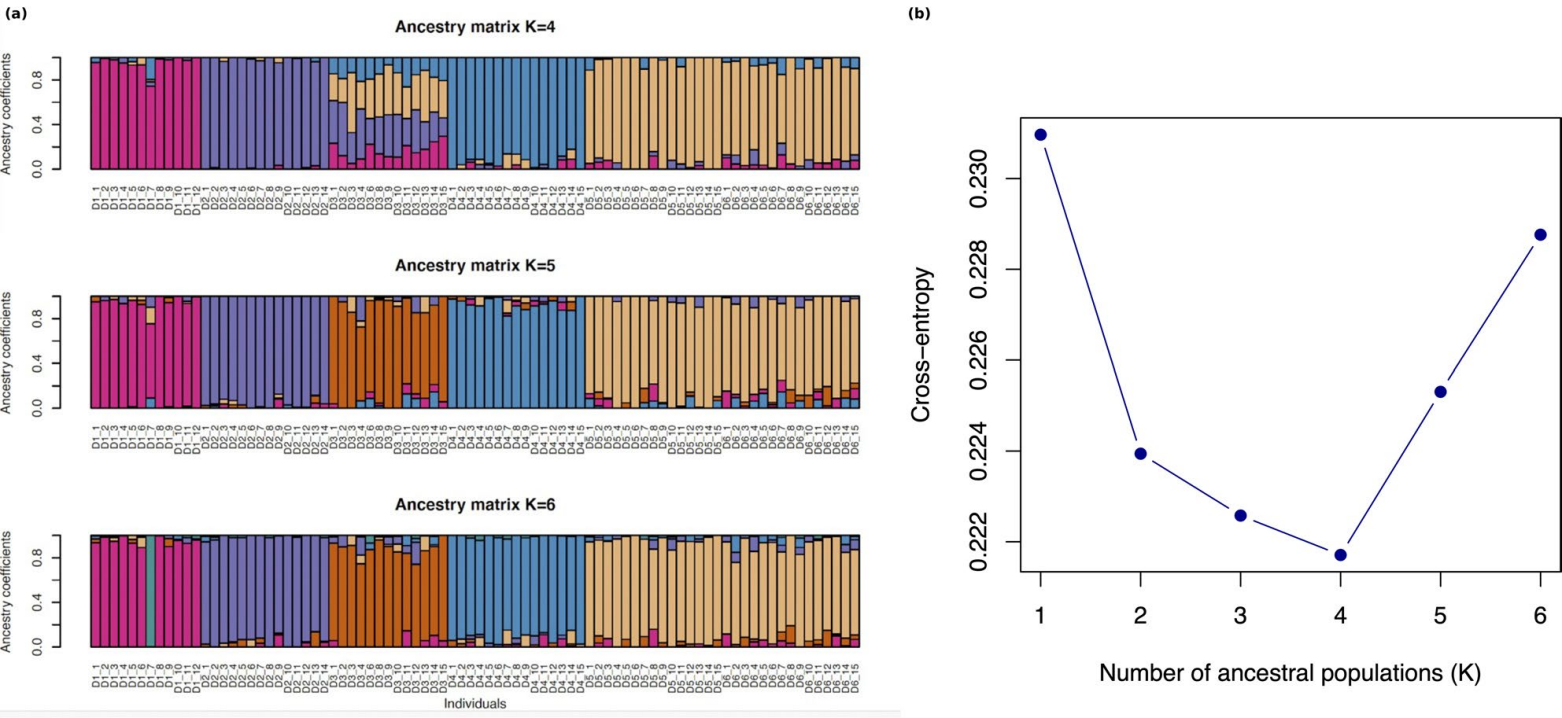
(**a**) sNMF bar graphs for K values 4 through 6. (**b**) The lowest CV error (e.g. “most probable” K) for K = 4, with K = 5 second. D1: shukoray, D2: Gali Colluli, D3: Zariga, D4: abaeded (*D. dinicolai*), D5: Afdera3 (*D. franchetti*), D6: Afdera8 (*D. franchetti*)

PCADAPT (Fig. 3) and sNMF (Fig. 4) analyses, conducted using the putatively unlinked SNP dataset, are largely concordant with the phylogenetic tree but also show some differences. Both the PCA and sNMF analyses indicate 4–5 genetic clusters corresponding to the three river populations (D1, D2, D3), and Lake Abaeded (D4) and Lake Afrera (D5, D6). The first two principal components show four clusters corresponding to the three rivers and with Lake Abaeded overlapping with the southern Lake Afrera samples, while the second and third principal components break out Abaeded as its own cluster for five clusters. sNMF results differ slightly: K = 4 and K = 5 are found to be the most likely number of clusters (with similar likelihoods across these two values; Fig. 4b); for K = 4, putative distinct populations correspond to Shukoray (D1), Gali Colluli (D2), and Abaeded (D4) (corresponding to the monophyletic clades with high support in the tree), with samples from Zariga (D3) showing shared ancestry with all other groups; when K = 5 is used, Zariga emerges as a population but showing admixture with other groups as before. These population-level analyses confirm the relative genetic isolation of Shukoray and Gali Colluli from other groups as indicated in the phylogenetic tree (Fig. 2). This differentiation is also reflected in the higher Fst values for these two populations compared with the other sample groups (Table 2). Both Abaeded and Zariga also show some population-level differentiation as shown in the PCA and sNMF analyses, and Fst values. In contrast, no differentiation between the two Afrera sampling locations is observed.

**Table 2:** Pairwise Fst values between sampled sites

	D2 (Gali Colluli)	D3 (Zariga)	D4 (Abaeded)	D5 (Afdera 3)	D6 (Afdera 8)
D1 (Shukoray)	0.0694986	0.0607185	0.0665019	0.0628529	0.0624173
D2 (Galli Colluli)	x	0.0566809	0.0649222	0.0605804	0.0608151
D3 (Zariga)		x	0.0524942	0.0478046	0.0485933
D4 (Abaeded)			x	0.0492256	0.0499006
D5 (Afdera 3)				x	0.0376782
D6 (Afdera 8)					x

## Discussion

The primary goal of this study was to better understand the history of diversification in the cichlid genus *Danakilia*, by assessing population structure and gene flow in the context of geological history. Our analysis represents the first investigation of this genus using genome-wide SNP data, and sheds new light on patterns of differentiation and gene flow in this clade.

Overall, despite the relatively young age of the habitat in which *Danakilia* evolved, genetic analyses indicate moderately high genetic divergence between many of the lineages of *Danakilia*. The geological record suggests that gene flow between the northern and southern Danakil depression may have last been possible during the humid mid-Holocene (7300–9800 YBP) when wetter conditions resulted in the formation of a paleo-lake that may have extended across most of the depression and was considerably less saline than at present [16, 23, 30–32]. In the northern Depression, higher water levels (about 50 m higher than present) may have facilitated the movement of fish into the Lake Abaeded crater, as the crater rim was then situated at least 20 m below the level of the paleo-lake [16]. With a subsequent decrease in rainfall after 7300 YBP, Lake Abaeded in the north became isolated, and Lake Afrera in the south rapidly receded to its present size of about 70 km^2^ [23] and to a salinity that is now roughly four times higher than seawater (130–159 ppt [15, 16]).

This geological history aligns with the genetic separation between the southern lineages that have been collectively called *D. franchettii*, and the monophyletic populations of the northern Danakil depression including *D. dinicolai* in Lake Abaeded and the three riverine populations (also supported by the morphological analysis of [17]). The topology of the phylogenetic tree (Fig. 2) indicates that *Danakilia* has had a longer continuous distribution in the southern portion of the depression, and likely colonized the northern habitats during the mid-Holocene when water levels were higher. While *D. dinicolai* of Lake Abaeded is sister to the riverine populations, we also observe strong differentiation between most of these northern populations (Figs. 2, 3, 4), consistent with the lack of any contemporary connections between the lake Abaeded and the nearby river networks (also demonstrated by the absence of dry riverbeds originating from the lake).

Interestingly, our analysis of genetic structure between the two *D. franchettii* populations sampled in springs (D5, D6) only partially supports the previous analyses that used concatenated mitochondrial genes. While Chiozzi et al. [16] indicated that the populations sampled in two springs are differentiated despite being only 5 km apart (noting, however, the low number of samples examined from each spring, *n* = 5), the ddRAD data indicate closer genetic similarity between the two locations, as demonstrated by the recovery of a single clade in the PCA (Fig. 3) and sNMF analyses for both the two best K values (Fig. 4). However, some minor genetic structure between the two populations is supported by an Fst value of 0.038 (Table 2). This low level of differentiation could result from ongoing, sporadic migration, suggesting that the extremely high salt concentration of lake waters may only form a partial barrier to movement of fish between different creeks. Movement between affluent creeks may be facilitated close to the shoreline where creeks with *Danakilia* have a salinity (average = 7.18 ppt, range = 4.41–10.34 ppt, *n* = 4) about 18 times lower than the lake (130 ppt) [16]. It seems reasonable to assume that inflowing creek waters with reduced salinity flow on top of the denser briny waters of the lake, create potential ecophysiological corridors for the movement of fish, albeit ephemeral ones, as complete mixing of layers occurs during frequent strong storms.

These data also shed some light on evolutionary relationships among the three riverine populations (Shukoray, Gali Colluli, and Zariga). The phylogenetic analysis (Fig. 2) indicates a sister relationship between these three populations, with samples from the three sites together forming a well-supported monophyletic group, as hypothesized given their geographic proximity (Fig. 1). The three riverine populations vary in the level of differentiation from one another and from other clades, as indicated by the tree (Fig. 2), PCA (Fig. 3), and by Fst values that are generally low to moderate (Table 2). Samples from Shukoray and Gali Colluli likewise are sister to one another and form well-supported clades (Fig. 2), whereas samples from Zariga are not monophyletic; however, all three populations are distinct in the PCA and sNMF plots at K = 5. The sNMF plots also suggest some level of historical gene flow between the three riverine populations and Lake Abaeded; additional demographic analyses are needed to test this possibility. This level of moderate differentiation is supported by some lines of morphological differentiation. Although Chiozzi et al. [17] were unable to identify attributes of body and lower pharyngeal jaw shape differentiating the riverine populations from *D. dinicolai* of Lake Abaeded, Chiozzi et al. [16] note that males of all riverine populations share a characteristic nuptial coloration of the dorsum and of the male nuchal hump distinct from that of either *D. franchettii* or *D. dinicolai* ([16]: Fig. 2a, b, c). This marked difference in reproductive coloration along with the overall result of this study suggest that the riverine populations may together represent at least one (and likely more) isolated lineage(s), and detailed comparative morphological studies are ongoing to locate other features (e.g., gut length and coiling pattern, oral dentition etc.) supporting the recognition of the riverine *Danakilia* as a species distinct from *D. dinicolai* (Chiozzi et al. in prep.).

Potential limitations of our study include unsampled populations. Due to logistical challenges and ongoing border military activities, sampling opportunities had been extremely limited, so we cannot rule out the existence of one or more “ghost” populations. Apart from the still unstudied population reported from the Ragali River, another population of *Danakilia* is potentially present in the northernmost tip of the Danakil Depression in a swampy area fed by permanent springs bordered with reeds and known as Lake Badda [33]. Since Lake Badda is situated in the same small endorheic basin of Lake Abaeded (Fig. 1), it could host a population genetically close to that of Lake Abaeded. Unfortunately, this hypothesis cannot be tested as access to Lake Badda is not currently possible. However, future research prioritizing sampling of populations from the Ragali River and an exploration of Lake Badda will be necessary to gain a more complete understanding of the ecological dynamics and phylogeography of the northern *Danakilia* populations. This is of particular importance from a conservation perspective as riverine populations throughout the northern Danakil will likely be deeply affected by potash mining, with huge extraction projects underway in both Ethiopia and Eritrea. While *Danakilia dinicolai* in Lake Abaeded seems to be safe from human disturbance for the time being, windblown sand from the surrounding desert drifts into the lake and could eventually eliminate this water body. Finally, the populations of *D. franchettii* are likely to be affected by extensive salt extraction from Lake Afrera, which has been expanding since the late 1990s [34] GC and GDM personal observation), has already destroyed habitats of *Danakilia franchettii,* and is lowering the salinity of the lake, with unknown consequences [17].

## Conclusions

The first genomic study on the cichlid genus *Danakilia* provides novel insights into their diversification history, likely influenced significantly by mid-Holocene climatic conditions and geological events. The expansion and subsequent recession of a mid-Holocene paleo-lake, coupled with geological and hydrological dynamics, have played a pivotal role in shaping population structure and gene flow between populations. Despite some genetic overlap between populations, our findings suggest that the hydrogeography of the region has resulted in genetic differentiation between sites, with notable differentiation of riverine populations. Our study also highlights conservation concerns on these little-known lineages due to anthropogenic impacts, emphasizing the need for further research on the ecological dynamics and phylogeography of the genus, particularly in light of the threats posed by industrial activities in the region [35, 36].

## Data Availability

Raw read data are archived in the NCBI Sequence Read Archive (BioProject: PRJNA1337280, Submission ID: SUB15681603).
